# Trigeminal Stimulation and Visuospatial Performance: The Struggle between Chewing and Trigeminal Asymmetries

**DOI:** 10.3390/biomedicines11082307

**Published:** 2023-08-19

**Authors:** Maria Paola Tramonti Fantozzi, Vincenzo De Cicco, Paola d’Ascanio, Enrico Cataldo, Davide De Cicco, Luca Bruschini, Massimo Barresi, Ugo Faraguna, Diego Manzoni

**Affiliations:** 1 Department of Translational Research and of New Surgical and Medical Technologies, University of Pisa, 56123 Pisa, Italy; 2 Department of Physics, University of Pisa, 56127 Pisa, Italy; 3 Department of Surgical, Medical and Molecular Pathology and Critical Care Medicine, University of Pisa, 56124 Pisa, Italy; 4 Department of Developmental Neuroscience, IRCCS Fondazione Stella Maris, 56128 Pisa, Italy

**Keywords:** chewing, locus coeruleus, trigeminal imbalance, bilateral and unilateral chewing, visuospatial performance

## Abstract

Chewing improves visuospatial performance through locus coeruleus (LC) activation. The effects of bilateral and unilateral mastication were investigated in subjects showing different degrees of asymmetry in masseter electromyographic (EMG) activity during clenching and in pupil size at rest (anisocoria), which is a proxy of LC imbalance. Correlations between performance changes and asymmetry values were found in males, but not in females. Among males, subjects with low asymmetry values (balanced-BAL) were more sensitive than those with high asymmetry values (imbalanced-IMB) to bilateral and unilateral chewing on the side with higher EMG activity (hypertonic). The opposite was true for hypotonic side chewing. BAL subjects were sensitive to unilateral chewing on both sides, while in IMB subjects, hypertonic side chewing did not influence performance in either males or females. Bilateral chewing elicited larger effects in BAL subjects than in IMB subjects, exceeding the values predicted from unilateral chewing in both groups. Finally, pupil size and anisocoria changes elicited by chewing were correlated with asymmetry values, independent of sex. Data confirmed the facilitation of visuospatial performance exerted by chewing. Trigeminal asymmetries modulate the chewing effects, making occlusal rebalancing an appropriate strategy to improve performance.

## 1. Introduction

Trigeminal nerve stimulation (TNS) has been utilized for clinical purposes, such as the treatment of drug-resistant epilepsy and refractory trigeminal neuralgia, as well as major depression, post-traumatic stress, and attention deficit hyperactivity disorders [1]. TNS activates the locus coeruleus (LC) [2], which enhances arousal and improves cognitive function [3,4,5]. This procedure may therefore improve cognitive performance, but the evidence for such effect is scant [1]. Recent studies addressing TNS effects on cognitive event-related potentials elicited by an oddball task have provided conflicting results [6,7], likely due to differences in the sensory stimulus (acoustic/visual) and/or the stimulated trigeminal branch (ophthalmic/mandibular). This is not the case with trigeminal stimulation that occurs during chewing activity, which is based on both motor and sensory signals. Chewing improves cognitive performance [8], memory and learning [9,10,11] (see however [12,13]), and reduces the visual reaction time of subjects [8]. These effects are coupled with an increase in cerebral blood flow in the brain regions engaged in the performed task [8]. Moreover, chewing shortens the latencies of event-related potentials evoked during an auditory oddball sound discrimination paradigm [14].

These chewing effects on cognitive performance might be related to the influence of chewing on alertness [15,16] and attention [17]. Recent investigations [18,19] suggest that sensorimotor trigeminal signals activate noradrenergic LC neurons implicated in arousal control [20,21]. Indeed, a chewing activity performed on both sides improved performance in a visuospatial task (the Spinnler–Tognoni numerical matrices test [22] for focused attention) and increased the mydriasis associated with execution of a haptic task [19]. The corresponding changes were significantly correlated. Due to the close correlation observed between LC activity and pupil size [5,23,24], these changes can be considered as a proxy of phasic LC activation during task execution [25,26,27].

While chewing enhances cognitive visuospatial performance, a trigeminal imbalance is detrimental. Indeed, in subjects showing an asymmetric development in their masseter electromyographic (EMG) activity during clenching, bite plate wearing improved both visuospatial performance and haptic task-related mydriasis [28,29,30,31]. Interestingly, bite correction also seemed to make the execution of skilled finger movements easier [32]. Also, the imbalance-elicited impairment in visuospatial performance seems to be related to central noradrenergic activity, as the subjects developing an asymmetric EMG activity during clenching also showed asymmetry in pupil size (anisocoria) at rest. In these subjects, the side with higher EMG activity (hypertonic side) corresponded to that with the larger pupil size. This anisocoria, which is suggestive of an imbalance in LC activity at rest, was decreased by bite plate wearing to the same extent as the EMG asymmetry [28,29,30]. The reasons why such an imbalance can be detrimental to performance have been previously addressed [28,29,30,33].

The stimulating effect of chewing on visuospatial cognition [19] is biased by the presence of sensorimotor trigeminal imbalance. In this respect, unilateral chewing improves performance in the Spinnler–Tognoni test only if performed on the side of lower (hypotonic) EMG activity during clenching [34]. Moreover, the efficacy of unilateral and bilateral chewing in subjects with different degrees of trigeminal asymmetry has not been clarified. 

The purpose of the present study was to assess, in the same population of subjects, the effects of bilateral and unilateral chewing on visuospatial performance and pupil size. Specifically, we aimed to study whether the effects of bilateral chewing could be predicted by the sum of the effects elicited by consecutive bouts of unilateral chewing on each side. 

Moreover, we investigated whether the effects of chewing on visuospatial cognition and pupil size were modulated by the degree of trigeminal imbalance, leading to a significant correlation between cognitive visuospatial improvement and EMG asymmetry.

## 2. Materials and Methods

### 2.1. Subjects

The experiments were performed on 30 healthy, right-handed subjects (age: 37.2 ± 14.0 years, 15 females), who did not complain of pain or discomfort during habitual masticatory activity. Five subjects (2 females) showed mild signs of temporomandibular disorders (TMDs) during the objective clinical evaluation (a click at initial mandible lowering and/or pain at extreme opening position).

### 2.2. Experimental Design

**Figure 1:**
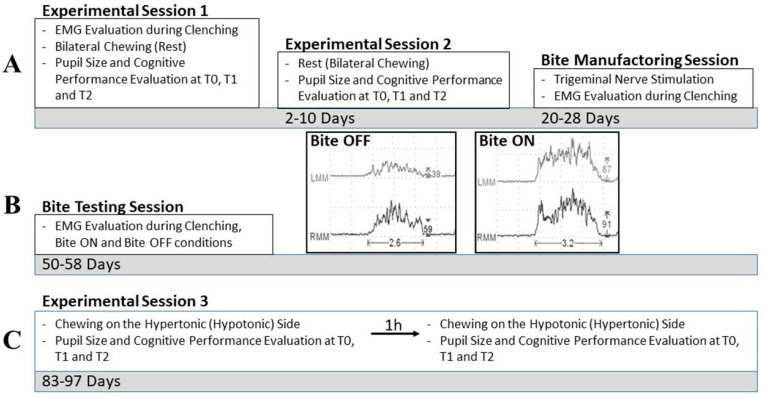
Flow chart of the experimental procedures. (**A**–**C**) represent successive periods of time. The 5 main boxes represent the performed sessions. The time passed from the initial session (experimental session 1) is indicated in the grey bars. The inner boxes report (from above) the execution of trigeminal nerve stimulation/EMG recordings, the specific chewing condition evaluated in the session, and the recorded variables measured at T0, T1, and T2. The insets in (**B**) represent the EMG activity recorded in a representative subject during the bite testing session, while clenching with (Bite ON) or without (Bite OFF) the bite splint interposed between the dental arches. Calibration bars along the X- and Y-axes report measures in seconds and µV, respectively. LMM: left masseter muscle. RMM: right masseter muscle.

This study analyzed the modifications in pupil size, anisocoria, and cognitive visuospatial performance elicited under four different experimental conditions, which were tested in three different experimental sessions. Moreover, between the second and third experimental session, subjects could be summited to two additional sessions for (1) manufacturing a bite splint and (2) testing its effectiveness in modifying EMG asymmetry. The flow chart of the whole experimental procedure is illustrated in Figure 1. 

In experimental session 1 (Figure 1A), as a preliminary step, all subjects underwent an evaluation of the average left and right masseter EMG activity observed during a clenching effort (Figure 1B, insets). In some subjects, the two values were comparable, while a substantial difference could be observed in others. We refer to the sides with higher and lower EMG activity during clenching as the hypertonic and hypotonic sides, respectively. 

The degree of asymmetry in the average EMG value during clenching (trigeminal asymmetry) was quantified by the difference in EMG activity between the hypertonic and hypotonic sides divided by the corresponding mean: (EMG hypertonic − EMG hypotonic)/((EMG hypertonic + EMG hypotonic)/2).

The participants showing masseter EMG asymmetry during clenching higher than 20% (n = 22, 15 females) were selected for an occlusal rebalancing procedure. We refer to these 22 participants as imbalanced (IMB) subjects. Twelve of them (10 females) showed higher EMG activity on the right side, while ten showed higher activity on the left side (5 females). Four of them (3 females) displayed mild signs of TMDs. The remaining 8 subjects with EMG asymmetry less than 20% were all males, and only one of them showed signs of TMDs. We refer to these participants as balanced (BAL) subjects.

Following this preliminary assessment, pupil size and performance-related variables were studied either at rest or under bilateral chewing conditions. Whatever the condition analyzed, the initial T0 baseline measurements consisted of:

(a) Bilateral recording of pupil size at rest;
(b) Performance assessment in a cognitive visual search task, based on the Spinnler–Tognoni numeric matrices test [19], in the resting mandible position.

These measurements took about five minutes and were followed either by a two minute period of hard pellet bilateral chewing [19] (bilateral chewing condition) or a two-minute rest period (rest condition). Chewing activity took place for one minute per side, with the starting side chosen by the subject. Subjects were invited to chew at their spontaneous rate, which was not controlled by the experimenter.

Measurements a and b were repeated soon after the bilateral chewing/rest period (T1), occurring approximately 7 min after T0 and followed by a further 30-minute period of rest (T2).

From 2 to 10 days later, in experimental session 2 (Figure 1A), subjects were submitted again to the same protocol according to a counterbalanced design where the participants who chewed in the previous session rested in the second session, and vice versa.

About 18 days after experimental session 2, the IMB subjects underwent the bite splint-manufacturing session (Figure 1A). According to the procedure utilized for preparing a dental power splint [35], the arches were positioned in myocentric occlusion [36], which was achieved by isotonic muscle contraction originating from the rest position with the arches apart. To avoid possible mandibular trajectory disturbances arising from asymmetries in muscle tone, electrical trigeminal nerve stimulation, which induced relaxation of the masticatory muscles, was performed [37] (see Section 2.5). Once relaxation of the masticatory muscles was achieved, the arches were brought into contact and an imprint of the occlusal condition was taken. When subjects clenched with this imprint in their mouth, the EMG activity during clenching became symmetric. Starting from this imprint, within a couple of weeks, a bite splint was manufactured. This bite splint covered the inferior dental arch, and the IMB subjects wore it for two weeks. Participants were invited to wear the bite splint all day and night, except when eating. They reported full compliance with this instruction. Following this two week period, subjects underwent a bite testing session (Figure 1B). In this session, the EMG evaluation during clenching was performed with (Bite ON) and without (Bite OFF) the interposition of the bite splint between the arches. These evaluations confirmed that the EMG asymmetry during clenching was reduced by bite splint wearing (from 43.35 ± 16.70% to 7.33 ± 3.43%, *p* = 0.001). An example of this reduction in trigeminal asymmetry is shown in the insets of Figure 1B.

Experimental session 3 (Figure 1C) began following an overall period of 48–54 days of bite splint wearing (80–86 days from experimental session 1). In this session, the effects of unilaterally chewing a hard pellet were assessed in all 30 subjects, according to the previously described procedure:

1. Baseline (T0) measurements (a, b);
2. Unilateral chewing on the right side (1 min);
3. T1 measurements (a, b);
4. T2 measurements (a, b).

After one hour of rest, the entire T0-T1-T2 sequence was repeated, inviting the subjects to chew a new pellet with the same characteristics as the previous pellet on the left side.

### 2.3. Evaluated Parameters

The analysis was based on the following parameters, evaluated at T0, T1, and T2:

(1) Bilateral pupil size at rest;
(2) Difference in pupil size (anisocoria)—this parameter could be calculated either as left–right or hypertonic–hypotonic side differences;
(3) Performance index (PI: target numbers retrieved in 15 s/15), this metric has been described in detail elsewhere [19,22]).

### 2.4. Numerical Matrices Test

Subjects sequentially scanned three (10 × 10) numerical matrices, retrieving and ticking with a pencil as many of the (target) numbers indicated above each matrix as possible [22]. To prevent learning processes, the position of each target number within the matrices presented were modified between time points.

### 2.5. Electrical Trigeminal Nerve Stimulation for Bite Splint Manufacturing

The left and right trigeminal mandibular nerves were activated by two separate couples of electrodes (IACER, I-Tech Medical Division, Martellago, Venice, Italia, stimulating surface: 164 mm^2^). The two electrodes of each couple were placed on the incisura sigmoidea and on the submental triangle. Biphasic (cathodal/anodal) current pulses (0.54 msec, 21–25 mA) were delivered at the frequencies of 0.618 Hz (incisura sigmoidea) and 40 Hz (submental triangle) by two independent IACER stimulators (Martellago, Venice, Italy), as described elsewhere [28]. This procedure elicited contraction/relaxation of the masseters and tonic activation of lowering muscles, leading to small-amplitude (1 mm) mandibular movements in the sagittal plane. The current intensity on each side was adjusted to obtain symmetric EMG responses.

### 2.6. Pupil Size Recordings

Pupil size measurements (mm) were performed using a corneal topographer-pupillographer (MOD i02, with chin support, CSO, Florence, Italy), which maintained constant artificial lighting of 40 lux over the eye and captured pictures of the eye surface. The iris image was acquired by the camera with an acquisition time of 33 msec. Pupil images and size measurements were displayed on the computer screen. The haptic task was based on a tangram, a puzzle of triangular, square, and parallelogram-shaped forms. Subjects were seated with their heads restrained by the pupillometer, with the dental arches apart. During the task, each subject received the parallelogram piece from the experimenter, which was to be repositioned in its original place using the dominant hand under tactile control. 

Left and right pupil measurements were collected during separate task repetitions at rest when the subject began to explore the puzzle surface. During this procedure, vision was occluded.

### 2.7. Pellet for Chewing

The pellet for chewing (OCM Projects, L’Aquila, Italy) had a cylindrical shape (1.0 × 1.0 × 1.5 cm), a reticular structure, and was made of silicon rubber (gls50, Prochima, Colli al Metauro, Pesaro-Urbino, Italy). Its hardness (60 Shore OO) was unmodified by chewing. It was gray in color, sugar-free, odor-free, and tasteless.

### 2.8. EMG Data

Masseter EMG activity was recorded using Duotrode Ag/AgCl electrodes (interelectrode distance: 19.5 mm, MyoTronics, Seattle, WA, USA), placed as previously described [28,38]. EMG activity was recorded by a K6-I MyoTronics system (sample rate: 720 Hz, cut-off frequency: 15 Hz, notch filter: 50 Hz). The filtered EMG traces were fully rectified. The EMG measurements were performed during the EMG burst produced by the subject when clenching was performed (Figure 1B) and corresponded to the average value of the EMG trace during the burst, i.e., the area under the EMG trace divided by the duration of the burst. Both burst duration and average EMG values were automatically evaluated by the instrument and outputted together with the EMG traces. The frequency parameters reflecting the firing of individual motoneurons [38] were not analyzed. The clenching effort began and ended upon experimenter request and lasted from 2 to 4 s.

### 2.9. Statistical Analysis

The distribution of the data was assessed using the Komolgorov–Smirnov test. When the data did not comply with a spherical distribution, the Huynh–Feldt correction was applied to the ANOVA output. The comparison between the changes in performance and pupil-related parameters elicited by the different chewing patterns at T1 and T2 with respect to T0 was performed using a 4 Conditions (bilateral chewing, chewing on the hypertonic side, chewing on the hypotonic side, rest) × 2 Times (T1, T2) repeated measures ANOVA. To assess the significance of the chewing-induced modifications on cognitive visuospatial performance and pupil size-related parameters, a 3 Times (T0, T1, T2) repeated measures ANOVA was applied for each condition. These analyses were applied to the whole population, as well as separately to males and females.

The changes observed in pupil size, anisocoria, and PI at T1 and T2 with respect to T0 were separately correlated with EMG asymmetry and anisocoria by linear regression analysis. Statistics were obtained for the whole population, as well as separately for males and females.

Finally, to verify the degree of coupling between changes in pupil size parameters and performance, differences between time points were evaluated (T1–T0, T2–T0, T2–T1) separately for each chewing condition (bilateral chewing, chewing on the hypertonic side, chewing on the hypotonic side) and submitted to linear regression analysis. Comparisons of slopes and correlation coefficients were also performed [39,40,41]. 

The significance level was set at 0.05 for PI. Since pupil size-related variables (pupil size on the hypertonic and hypotonic sides, anisocoria) could be interdependent, Bonferroni’s correction was applied and the significance level was set at 0.05/3, i.e., to 0.017. 

IBM SPSS Statistics for Windows version 20.0 (IBM Corp. Released 2011. Armonk, NY, USA) was used for the analysis.

## 3. Results

### 3.1. Baseline Values of EMG and Pupil Size Asymmetries 

**Figure 2:**
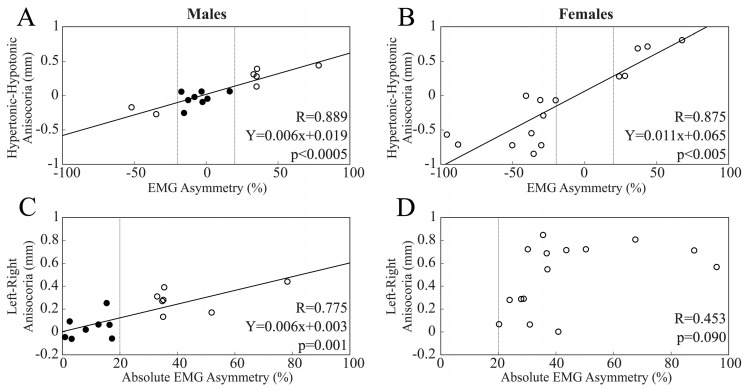
Correlation between anisocoria and EMG asymmetry. Anisocoria and EMG asymmetry evaluated as left–right side difference (**A**,**B**) as well as hypertonic–hypotonic side difference (**C**,**D**). Males and females are plotted in (**A**,**C**) and (**B**,**D**), respectively. Anisocoria baseline values are the averages evaluated across conditions (n = 4) at T0. For (**A**–**C**), continuous lines correspond to the regression lines evaluated across all of the plotted points. Vertical dotted lines separate BAL (dots) from IMB (circles) subjects.

Average baseline anisocoria and EMG asymmetry values (evaluated as hypertonic–hypotonic side difference) corresponded to 0.32 ± 0.29 mm and 34.6 ± 23.5%, respectively. Average anisocoria (0.49 ± 0.29 mm) and EMG asymmetry values (43.8 ± 22.7%) observed in females were significantly higher than those observed in males (anisocoria: 0.15 ± 0.16 mm, *p* = 0.001; EMG asymmetry: 25.3 ± 21.10%, *p* = 0.028). This finding was attributable to the absence of females showing symmetric EMG activity during clenching (BAL subjects) and the positive correlation between anisocoria and EMG activity. Indeed, when pupil size and EMG asymmetries were expressed as left–right difference, a positive correlation was observed between these two parameters, with the larger pupil size being located on the side with higher EMG activity. Although the data from the whole population could be well fitted by a single regression line (R = 0.859, Y = 0.009X + 0.019, *p* < 0.0005), a separate analysis of males (R = 0.889, Y = 0.006X + 0.019, *p* < 0.0005, Figure 2A) and females (R = 0.876, Y = 0.011X + 0.065, *p* < 0.0005, Figure 2B) revealed a significantly higher slope for the latter (t = 2.236, *p* = 0.034). When anisocoria and EMG asymmetry values were evaluated as hypertonic–hypotonic side difference (Figure 2C,D), the two parameters were still significantly correlated in males (R = 0.775, Y = 0.006X + 0.003, *p* = 0.001) but not in females (R = 0.453, *p* = 0.090). 

### 3.2. Time Course of Chewing Effects on Pupil and Performance Parameters 

The effects of the different chewing patterns and rest period on visuospatial performance and pupil size parameters were assessed using a 3 Times (T0, T1, T2) repeated measures ANOVA, which was separately applied for each condition. The results of this analysis are summarized in Table 1.

While simple repetitions of the measurement did not affect the PI, PI values increased significantly at T1 for all chewing patterns, although the changes elicited by chewing on the hypertonic side were very small. However, the effect persisted at T2 only for bilateral chewing. No between-gender differences were found under the conditions of bilateral chewing and chewing on the hypotonic side. Following chewing on the hypertonic side, a significant time effect was observed only for males (F(2,28) = 15.67, *p* < 0.0005). In this group, PI values at T1 (1.68 ± 0.37 Nos./s) were significantly larger than the T0 (1.58 ± 0.33 Nos./s, *p* < 0.0005) values.

Pupil size values on both sides were not affected by significant spontaneous fluctuations, as indicated by the lack of a significant time effect in the rest condition (Table 1). Following bilateral chewing, pupil size increased on the hypotonic side, while decreasing on the hypertonic side, with both changes being significant at T1. Following chewing on the hypertonic side, bilateral and significant increases in pupil size were observed at T1 and T2. Chewing on the hypotonic side led to similar effects, but the increase observed at T2 on the hypotonic side was not significant. Both males and females showed similar behaviors. Although the time effect (F(2,58) = 3.711, *p* = 0.030) observed for anisocoria in the rest condition was not present after Bonferroni’s correction, the post hoc comparison indicated a significant difference at T1 with respect to T0. This increase in anisocoria values at T1 was exclusively due to females showing a significant time effect (F(2,28) = 9.971, *p* = 0.001), with the T1 values (0.53 ± 0.28 mm) being significantly higher with respect to both the T0 (0.42 ± 0.25 mm, *p* = 0.001) and T2 (0.47 ± 0.24 mm, *p* = 0.024) values. At variance, neither a significant time effect (F(2,28) = 1.228, *p* = 0.308) nor significant differences between the T0 (0.11 ± 0.19 mm), T1 (0.15 ± 0.22 mm), and T2 (0.06 ± 0.24 mm) time points could be observed in the male population. Bilateral chewing elicited a significant drop in anisocoria values both at T1 and T2 with respect to T0, and the same occurred following unilateral chewing on the hypotonic side, but in this instance, anisocoria values at T2 and T0 did not differ. At variance, chewing on the hypertonic side significantly increased anisocoria values at both T1 and T2 (Table 1). For all chewing patterns, the behaviors of males and females were similar.

### 3.3. Comparison of Changes in PI and Pupil Size Elicited under Different Conditions

Changes in performance and pupil size parameters at the different times and under different conditions were analyzed using 4 Conditions × 3 Times repeated measures ANOVA. Significant condition × time effects were observed for PI (F(3,87) = 8.86, *p* = 0.001), hypertonic side pupil (F(3,87) = 4.75, *p* = 0.008), hypotonic side pupil (F(3,87) = 22.47, *p* < 0.0005), and anisocoria (F(3,87) = 32.20, *p* < 0.0005), which are decomposed in Table S1. It was observed that bilateral chewing elicited a larger PI change with respect to unilateral chewing on either side, both at T1 and T2. T1 changes were always larger than T2 changes. It was noticed, however, that only the changes related to bilateral (T1 and T2) and unilateral hypotonic side chewing (only at T1) were significantly larger than those occurring by chance (rest condition). Males and females showed similar behaviors with respect to conditions and times.

At T1, bilateral chewing had opposite effects on the size of the hypertonic (reduction) and hypotonic (increase) side pupils, while unilateral chewing always increased pupil size bilaterally, although to a larger extent on the ipsilateral side. Contralateral pupil size changes elicited by unilateral chewing were not significantly different from those observed in the rest condition. Pupil size changes at T2 were not significantly different from those at T0 and they were not significantly different from those observed in the rest condition. Finally, the decreases in anisocoria values elicited at T1 by bilateral and unilateral chewing on the hypotonic side were significantly larger than those in the rest condition, while this was not the case for the increase in anisocoria values elicited by unilateral chewing on the hypertonic side. At T2, only the anisocoria changes elicited by bilateral chewing were significantly larger with respect to the rest condition.

An attempt was made to verify whether PI changes between time points could be predicted by the corresponding pupil size and anisocoria changes. This analysis focused on those changes in pupil parameters that were significantly different from the spontaneous fluctuations observed in the rest condition (Table S1). Moreover, the correlation with anisocoria changes was analyzed only among IMB subjects who showed sizeable values (and changes) of this parameter. Under the hypotonic side chewing condition, changes in hypotonic side pupil size were highly predictive of changes in PI (R = 0.751, Y = 0.588X + 0.008, *p* < 0.0005). Similar findings were obtained in the different gender and asymmetry (BAL, IMB) groups. A strong negative correlation was also observed between PI and anisocoria changes (R = 0.754, Y = −0.683 + 0.031, *p* < 0.0005), with similar results observed in males and females.

Under the hypertonic side chewing condition, a correlation was observed between PI and hypertonic side pupil changes (R = 0.439, Y = 0.277X − 0.001, *p* < 0.0005). This correlation, however, was entirely due to the 8 BAL male subjects (R = 0.843, Y = 0.666X − 0.012, *p* < 0.0005), since it was not observed among the IMB subjects (R = 0.209, *p* = 0.092), irrespective of their gender. It was also observed when only males (BAL and IMB) were taken into account (R = 0.603, Y = 0.394X − 0.006, *p* < 0.0005).

Under the bilateral chewing condition, a loose correlation was observed between PI and pupil size changes (hypertonic side: R = 0.232, Y = −0.410X + 0.286, *p* = 0.028; hypotonic side: R = 0.237, Y = 0.420X + 0.286, *p* = 0.025). While males and females showed the same trend for the hypertonic side pupil, the correlation observed for the hypotonic side pupil was exclusively due to the females (R = 0.526, Y = 0.748X + 0.238, *p* < 0.0005), while the males did not show any trend between these parameters (R = 0.055, *p* = 0.812). When BAL and IMB subjects were taken into account, this correlation was only observed among IMB subjects. Under the bilateral chewing condition, PI changes were also negatively correlated with anisocoria changes in IMB subjects (R = 0.502, Y = −0.539X + 0.225, *p* < 0.0005). This correlation was mainly due to the female group (R = 0.586, Y = −0.596X + 0.185, *p* < 0.0005), with males showing a similar but non-significant slope (R = 0.224, *p* = 0.328). 

### 3.4. Bilateral Chewing: “Experimental” and “Predicted” Effects on Cognitive Visuospatial Performance

In the present study, unilateral chewing lasted for one minute, whatever the side, while bilateral chewing consisted of two chewing bouts of one minute (one on the hypertonic side and one on the hypotonic side). For this reason, we investigated whether the effects of bilateral chewing on PI could be predicted by summating those of unilateral chewing. The changes induced by bilateral chewing at T1 with respect to T0 (0.62 ± 0.25, SD, Nos./s) were significantly higher than those obtained by summing the effects of unilateral chewing (0.23 ± 0.12, Nos./s, *p* < 0.0005). This held true for both males and females.

Within the whole population, the difference between the experimental and predicted values of PI change was loosely correlated with EMG asymmetry (R = 0.389, Y = −0.005X + 0.567, *p* = 0.034) but not to anisocoria baseline values (R = 0.212, *p* = 0.260). A more careful analysis revealed that females did not show a correlation of the experimental-predicted difference in PI change with EMG asymmetry (R = 0.019, *p* = 0.945) and anisocoria (R = 0.378, *p* = 0.316), while males did show these correlations (EMG asymmetry: R = 0.616, Y = −0.010X + 0.710, *p* = 0.014; anisocoria: R = 0.683, Y = −1.461X + 0.679, *p* = 0.005). The difference between R values of males and females was significant for EMG asymmetry (z = −1.713, *p* = 0.043). 

The size of the hypertonic side pupil increased following both conditions of unilateral chewing, while it decreased following bilateral chewing; consequently, the predicted values (0.14 ± 0.06 mm) were significantly higher than the experimental ones (−0.18 ± 0.7 mm, *p* < 0.0005). The same held true for the hypotonic side pupil, the size of which was increased under all chewing conditions (predicted: 0.31 ± 0.10 mm, experimental: 0.14 ± 0.22 mm, *p* < 0.0005).

### 3.5. PI Changes as a Function of EMG Asymmetry

**Figure 3:**
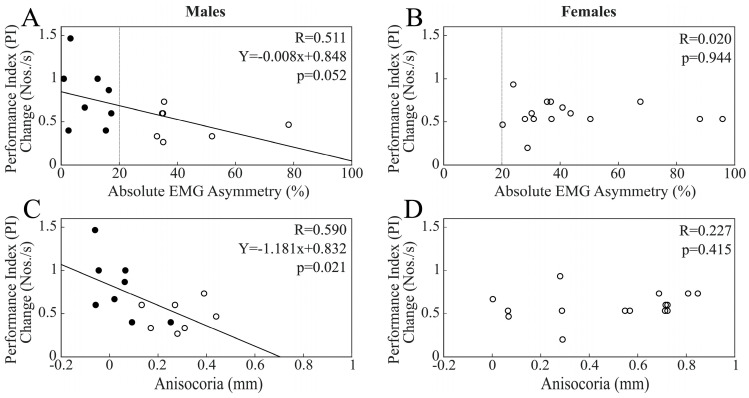
Scatterplots of changes in PI elicited at T1 by a period of bilateral chewing and measures of EMG (**A**,**B**) and pupil size (**C**,**D**) asymmetries. (**A**,**C**) and (**B**,**D**) refer to males and females, respectively. Continuous lines represent the regression lines evaluated for all of the plotted points, the equations of which are reported in the panels. Dots and circles refer to BAL and IMB subjects, respectively. The vertical dotted lines pass through the 20% EMG asymmetry value, which separates these two populations.

As a preliminary step, we controlled whether spontaneous PI changes occurring at T1 and T2 in the rest condition could be correlated with the baseline values of EMG asymmetry and anisocoria. No significant correlations were found at either time point in the whole population, nor separately among males and females. When the data from all subjects were pooled together, no significant correlation could be found between the changes in PI elicited by bilateral chewing at both T1 and T2 and EMG asymmetry. However, as shown in Figure 3A, PI changes elicited at T1 were quasi-significantly correlated with baseline values of EMG asymmetry in males, with the largest increases in PI change occurring in those subjects characterized by the lowest trigeminal imbalance. 

**Figure 4:**
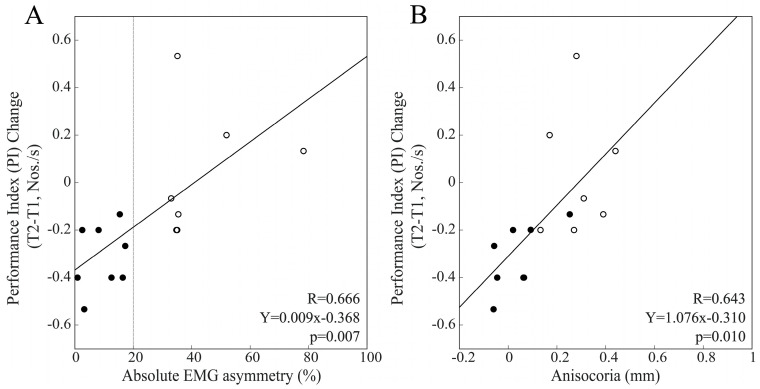
Scatterplots of T2–T1 PI changes elicited following bilateral chewing and measures of EMG (**A**) and pupil size (**B**) asymmetries in the male population. Continuous lines represent the regression lines evaluated for all of the plotted points, the equations of which are reported in each panel. Dots and circles refer to BAL and IMB subjects, respectively. The vertical dotted line passes through the 20% EMG asymmetry value, which separates these two populations.

This finding was supported by the significant difference in PI change (*p* = 0.05) observed between BAL (0.80 ± 0.36 Nos./s, n = 8) and IMB (0.48 ± 0.17 Nos./s, n = 7) subjects. Figure 3C shows the significant correlation existing between these PI changes and baseline values of anisocoria. Figure 3B,D shows the lack of correlation between changes in PI at T1 and asymmetry indices observed in the female population. PI changes at T2 were not correlated with EMG asymmetry, neither in males, nor in females. The loss of the correlation between PI changes and EMG/pupil size asymmetries observed at T2 among males was due to the larger drop in PI from T1 to T2 occurring among BAL subjects (−0.32 ± 0.14 Nos./s) with respect to IMB males (0.04 ± 0.27 Nos./s, *p* = 0.006). Figure 4 shows that in males, because of this difference, the changes in PI from T1 to T2 were significantly and positively correlated with both EMG asymmetry and anisocoria.

**Figure 5:**
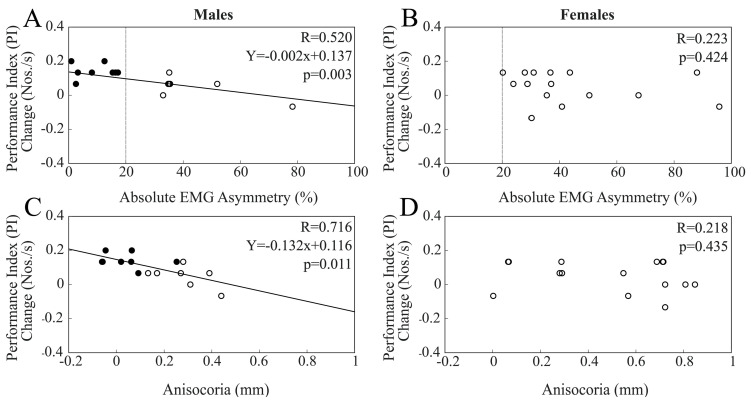
Scatterplots of changes in PI elicited at T1 by a period of unilateral chewing on the hypertonic side and measures of EMG (**A**,**B**) and pupil (**C**,**D**) asymmetries. (**A**,**C**) and (**B**,**D**) refer to males and females, respectively. Continuous lines represent the regression lines evaluated for all of the plotted points, the equations of which are reported in the panels. Dots and circles refer to BAL and IMB subjects, respectively. The vertical dotted lines pass through the 20% EMG asymmetry value, which separates these two populations.

When chewing on the hypertonic side was considered, changes in PI at T1 were significantly and negatively correlated with EMG asymmetry (R = 0.520, Y = −0.002X + 0.137, *p* = 0.003). The same results could also be observed for pupil asymmetry (R = 0.459, Y = −0.132X + 0.116, *p* = 0.011). As shown in Figure 5, these correlations reached significance only within the male population (Figure 5A,C), with the difference between R values being significant for both EMG asymmetry (z = −1.999, *p* = 0.023) and anisocoria (z = −1.681, *p* = 0.046). 

Interestingly, among males, the T1 and T0 PI values were significantly different for BAL subjects (T0: 1.65 ± 0.38 Nos./s; T1: 1.79 ± 0.40 Nos./s, *p* < 0.0005) but not for IMB subjects (T0: 1.50 ± 0.28 Nos./s; T1: 1.54 ± 0.30 Nos./s, *p* = 0.094). Indeed, PI changes at T1 in BAL males (0.142 ± 0.043 Nos./s) were larger than those in IMB males (0.048 ± 0.063 Nos./s, *p* = 0.005) and females (0.049 ± 0.089 Nos./s, *p* = 0.003). 

**Figure 6:**
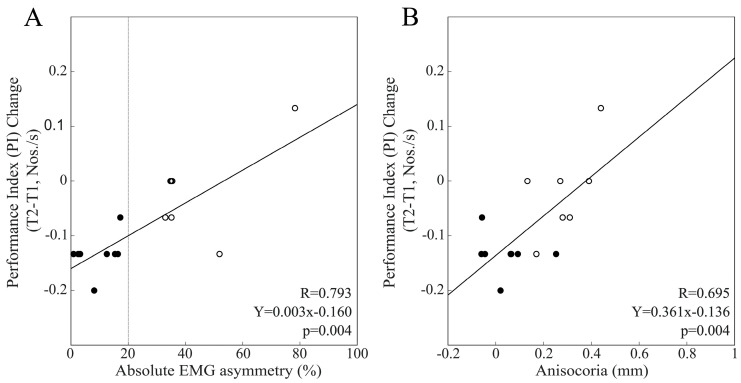
Scatterplots of T2–T1 PI changes elicited following unilateral chewing on the hypertonic side and measures of EMG (**A**) and pupil size (**B**) asymmetries in the male population. Continuous lines represent the regression lines evaluated for all of the plotted points, the equations of which are reported in the panels. Dots and circles refer to BAL and IMB subjects, respectively. The vertical dotted line passes through the 20% EMG asymmetry value, which separates these two populations.

No correlation was found between PI changes at T2 and EMG asymmetry, whatever population was considered. The lack of significant correlations among males could be attributed to the larger drop in PI at T2 with respect to T1 in the population of BAL subjects (−0.133 ± 0.036 Nos./s) with respect to IMB males (−0.02 ± 0.08 Nos./s, *p* = 0.004), leading to the significant correlation between the T2–T1 PI difference with EMG asymmetry and anisocoria (Figure 6). 

The T2–T1 PI difference in BAL males was also significantly different than that of IMB females (0.027 ± 0.094 Nos./s, *p* = 0.001). 

**Figure 7:**
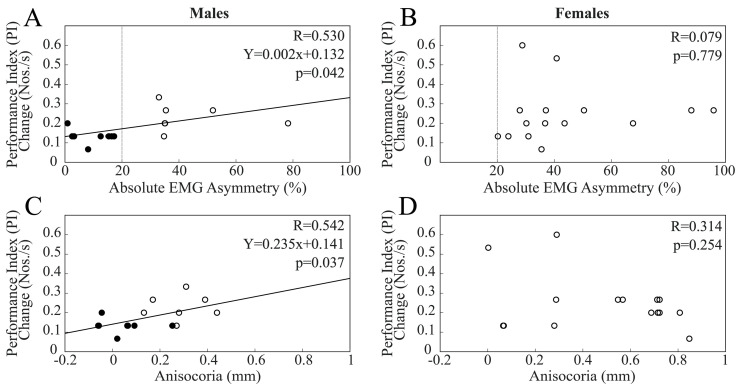
Scatterplots of changes in PI elicited at T1 by a period of unilateral chewing on the hypotonic side and measures of EMG (**A**,**B**) and pupil size (**C**,**D**) asymmetries. (**A**,**C**) and (**B**,**D**) refer to males and females, respectively. Continuous lines represent the regression lines evaluated for all of the plotted points, the equations of which are reported in the panels. Dots and circles refer to BAL and IMB subjects, respectively. The vertical dotted lines pass through the 20% EMG asymmetry value, which separates these two populations.

Finally, when chewing occurred on the hypotonic side, changes in PI at T1 were not significantly correlated with baseline EMG asymmetry (R = 0.299, *p* = 0.108) and anisocoria (R = 0.073, *p* = 0.701) values within the whole population. As shown in Figure 7, these correlations were present in males, but not in females. As observed for the other chewing patterns, PI changes at T1 were also correlated with anisocoria within the male population. 

Among males, PI changes in BAL subjects (0.133 ± 0.036 Nos./s) were smaller than those in IMB subjects (0.242 ± 0.133 Nos./s, *p* = 0.021), with the T1 PI values larger than T0 values in both BAL (T0: 1.69 ± 0.36 to 1.83 ± 0.37 Nos./s, *p* < 0.0005) and IMB (from 1.50 ± 0.28 Nos./s, T1: 1.54 ± 0.30 Nos./s, *p* < 0.0005) subjects. The T1–T0 PI difference (0.249 ± 0.144 Nos./s) was significant also among females (T0 PI values: 1.86 ± 0.57 Nos./s; T1 PI values: 2.11 ± 0.58 Nos./s, *p* < 0.0005), all of whom being IMB subjects.

No correlation was found between PI changes at T2 and asymmetry measures, whatever population was considered. 

### 3.6. Changes in Pupil Size Parameters as a Function of EMG Asymmetry

The spontaneous fluctuations in pupil size and anisocoria observed in the rest condition at T1 with respect to T0 were not correlated with baseline values of EMG asymmetry and anisocoria, whatever population was considered. This finding reinforced the results of the regression analysis performed on the pupil size and anisocoria changes elicited by the different chewing conditions. Only changes significantly larger than those elicited in the rest condition were analyzed. Pupil size changes elicited by bilateral chewing on each side were not correlated with baseline values of EMG asymmetry within the whole population, nor among males and females. A loose correlation was found between the changes on the hypertonic side and baseline values of anisocoria (R = 0.381, Y = −0.321X − 0.020, *p* = 0.038), with similar but insignificant trends observed in the two gender groups. A similar result was observed for the hypotonic side pupil, but in this instance, pupil size changes and anisocoria values showed a positive correlation (R = 0.485, Y = 0.375X + 0.020, *p* = 0.007).

Ipsilateral pupil size changes elicited by hypertonic side chewing were not correlated with EMG asymmetry, whatever population was considered (males, females, all subjects). A loose correlation was observed with baseline values of anisocoria (R = 0.366, Y = −0.101X + 0.213, *p* = 0.047), with similar negative trends in both males and females.

**Figure 8:**
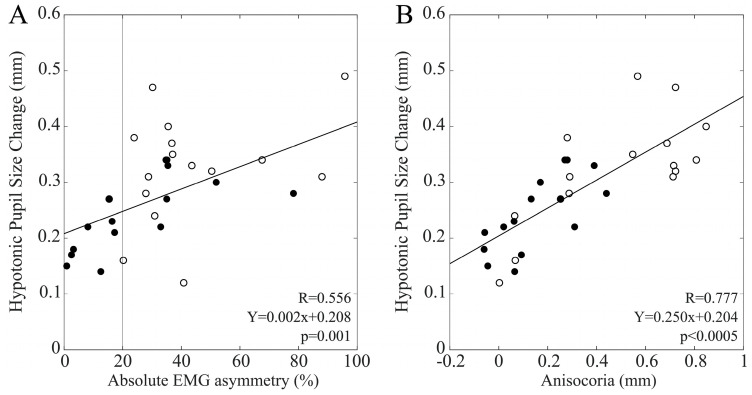
T1 hypotonic pupil size changes induced by a period of unilateral chewing on the same side as a function of baseline measures of EMG (**A**) and pupil size (**B**) asymmetries. Dots and circles refer to males and females, respectively. Continuous lines represent the regression lines evaluated for all of the plotted points, the equations of which are reported in the panels. The vertical dotted line in A passes through the 20% EMG asymmetry value, which separates BAL from IMB subjects.

As shown in Figure 8A, chewing on the hypotonic side elicited T1 pupil size changes on the ipsilateral side (hypotonic side) that were significantly correlated with EMG asymmetry in the whole population (R = 0.556, Y = 0.002X + 0.208, *p* = 0.001). Separate analyses of males and females indicated similar trends in the two groups, which reached the significance level in males (R = 0.676, Y = 0.002X + 0.189, *p* = 0.006) but not in females (R = 0.335, *p* = 0.222).

Figure 8B shows that these pupil size changes were also significantly correlated with baseline anisocoria values in the whole population (R = 0.777, Y = 0.250X + 0.204, *p* < 0.0005), as well as among males (R = 0.725, Y = 0.300X + 0.197, *p* = 0.002) and females (R = 0.712, Y = 0.241X + 0.207, *p* = 0.003).

**Figure 9:**
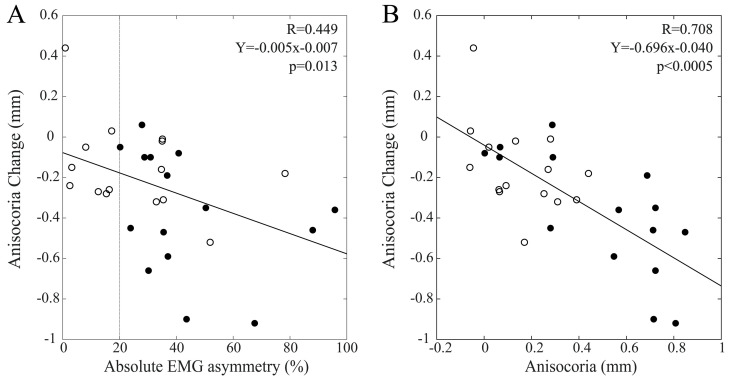
Scatterplots of changes in anisocoria elicited at T1 by a period of bilateral chewing and baseline measures of EMG (**A**) and pupil size (**B**) asymmetries. Dots and circles refer to males and females, respectively. Continuous lines represent the regression lines evaluated for all of the plotted points, the equations of which are reported in the panels. The vertical dotted line in A passes through the 20% EMG asymmetry value, which separates BAL from IMB subjects.

Significant anisocoria changes were elicited at T1 by bilateral chewing and by chewing on the hypotonic side. Figure 9A shows that the changes elicited by bilateral chewing were correlated with EMG asymmetry within the whole population (R = 0.449, Y = −0.005X − 0.077, *p* = 0.013). Similar but non-significant trends were observed among males (R = 0.363, *p* = 0.184) and females (R = 0.336, *p* = 0.220). A strong correlation between these anisocoria changes and the corresponding baseline values was observed within the whole population (R = 0.708, Y = −0.696X − 0.40, *p* < 0.0005), with males (R = 0.443, *p* = 0.098) and females (R = 0.725, *p* = 0.002) showing similar trends. These data are illustrated in Figure 9B.

**Figure 10:**
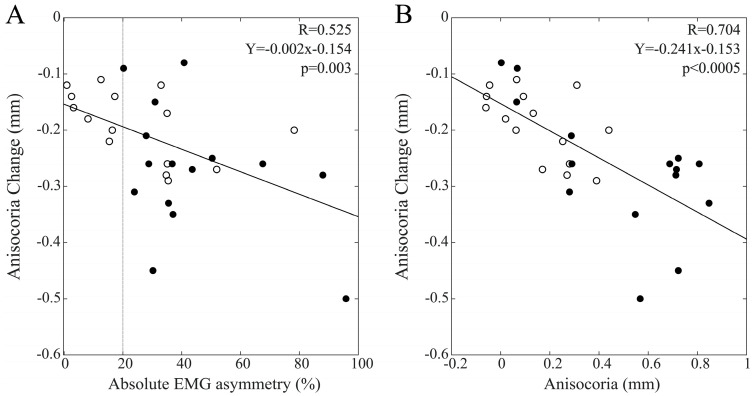
Scatterplots of changes in anisocoria elicited at T1 by a period of unilateral chewing on the hypotonic side and baseline measures of EMG (**A**) and pupil size (**B**) asymmetries. Dots and circles refer to males and females, respectively. Continuous lines represent the regression lines evaluated for all of the plotted points, the equations of which are reported in the panels. The vertical dotted line in A passes through the 20% EMG asymmetry value, which separates BAL from IMB subjects.

Finally, anisocoria changes elicited by chewing on the hypotonic side were also correlated with baseline values of EMG asymmetry (R = 0.525, Y = −0.002X − 0.154, *p* = 0.003), with both males (R = 0.504, *p* = 0.055) and females (R = 0.419, *p* = 0.120) showing similar trends. Anisocoria changes were negatively correlated with baseline anisocoria values within the whole population (R = 0.704, Y = −0.242X −0.153, *p* < 0.0005), with similar trends among males (R = 0.574, *p* = 0.024) and females (R = 0.644, *p* = 0.010). These data are summarized in Figure 10.

### 3.7. Differences in Performance Index Changes Elicited by the Different Chewing Patterns and Baseline EMG Asymmetry

**Figure 11:**
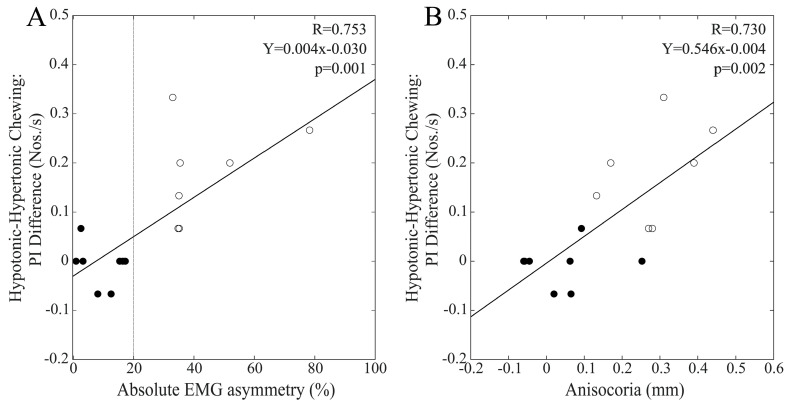
Relative efficacy of hypotonic and hypertonic side chewing in facilitating cognitive visuospatial performance as a function of baseline values of EMG asymmetry and anisocoria in the male population. The differences in PI change elicited by hypotonic and hypertonic side chewing are plotted as a function of baseline values of EMG asymmetry (**A**) and anisocoria (**B**). Continuous lines correspond to the regression lines for all of the plotted points, the equations of which are displayed in the corresponding panel. Dots and circles refer to BAL and IMB subjects, respectively. The vertical dotted line in (**A**) passes through the 20% EMG asymmetry value, which separates BAL from IMB subjects.

To assess whether the relative efficacy of the chewing patterns in modulating performance and pupil size was affected by the baseline values of EMG and pupil size asymmetries, we calculated the differences in performance and pupil size changes at T1 under the different chewing (bilateral, hypertonic side, hypotonic side) conditions. When the whole population was considered, the hypotonic–hypertonic side difference in PI change was significantly correlated with baseline EMG asymmetry (R = 0.474, Y = 0.003X + 0.025, *p* = 0.008) but not anisocoria values (R = 0.283, *p* = 0.130). As shown in Figure 11, this difference in PI change was strongly correlated with baseline values of both EMG asymmetry and anisocoria among males, while no correlation was found among females (EMG asymmetry: R = 0.163, *p* = 0.562; anisocoria: R = 0.149, *p* = 0.595). The differences in R values between males and females were significant for both EMG asymmetry (z = 1.997, *p* = 0.023) and anisocoria (z = 1.907, *p* = 0.028).

**Figure 12:**
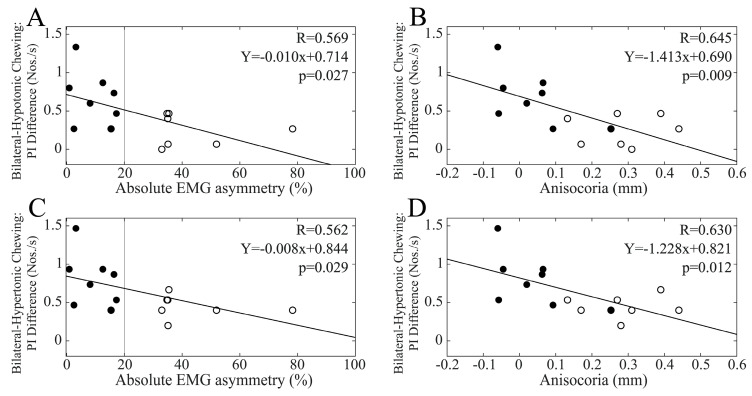
Relative efficacy of bilateral and hypotonic side chewing in facilitating cognitive visuospatial performance as a function of baseline values of EMG asymmetry and anisocoria. The bilateral–hypotonic (**A**,**B**) and bilateral–hypertonic (**C**,**D**) side chewing differences in PI change are plotted as a function of baseline values of EMG asymmetry (**A**,**C**) and anisocoria (**B**,**D**). The continuous lines correspond to the regression line of all the plotted points, the equations of which are displayed in the corresponding panel. The vertical dotted line in (**A**,**C**) passes through the 20% EMG asymmetry value, which separates BAL from IMB subjects. Dots and circles refer to BAL and IMB subjects, respectively.

When the whole population was considered, the bilateral–hypotonic side difference in PI change was significantly correlated with baseline EMG asymmetry (R = 0.379, Y = −0.005X + 0.583, *p* = 0.039) but not with anisocoria values (R = 0.188, *p* = 0.319). While females did not show any correlation of this relative performance indicator with the two measures of asymmetry (EMG asymmetry: R = 0.049, *p* = 0.862; anisocoria: R = 0.311, *p* = 0.260), strong correlations were observed among males for both EMG asymmetry and anisocoria (Figure 12A,B). 

Finally, when the bilateral–hypertonic side chewing difference in PI change was considered within the whole population, this parameter was not correlated with either EMG asymmetry (R = 0.345, *p* = 0.062) or anisocoria baseline values (R = 0.230, *p* = 0.222). However, analysis of the two gender groups revealed strong correlations among males with both EMG asymmetry and anisocoria (Figure 12C,D). At variance, females did not show any correlation with EMG asymmetry (R = 0.026, *p* = 0.926) or anisocoria (R = 0.166, *p* = 0.554). 

## 4. Discussion

### 4.1. General Considerations

Although the anatomical and physiological bases of the trigeminal effects on arousal have been assessed mainly in animal models [18], their impact on behavior has been extensively investigated in humans [8,12,13,15,16,17] and the present study lies within this field. While there is a large body of evidence about the detrimental effects of loss of masticatory function on cognitive processes [18], the influence of trigeminal asymmetries and their interaction with chewing-elicited arousal is less understood [34].

It is unknown, at present, whether there is a correspondence between the asymmetry that can be observed in force development during clenching and masticatory predominance, which is defined as the between-side difference in the occurrence of chewing strokes divided by the total number of strokes. It must be pointed out that, during gum chewing, about half of normal subjects show a masticatory predominance larger than 20% [42]. As previously documented [28,29,30], subjects with EMG asymmetry during clenching also show asymmetry in pupil size (anisocoria) at rest, with the larger pupil size occurring on the hypertonic side. Neither EMG nor pupil size asymmetries are associated with specific occlusal pathologies. Since pupil size is considered a proxy of LC activity [5,23,24,25,26,27], it has been proposed that asymmetry in sensorimotor trigeminal activity during clenching may lead to asymmetric activity at rest in the pathways linking trigeminal afferents to the LC [18], and, as a consequence, to asymmetric LC activity [30]. The crossed projections between the two LC complexes [43], which are likely to exert an inhibitory action on the target neurons [44,45], may enhance the imbalance elicited by asymmetric trigeminal input. The present findings suggest that the effectiveness of trigeminal imbalance in modifying the activity of central pathways controlling pupil size is different in males and females. Indeed, a given amount of EMG asymmetry is predictive of a larger anisocoria in females with respect to males, while the amount of this asymmetry accounts for a larger fraction of anisocoria variability in males (60%) with respect to females (21%), as indicated by the corresponding coefficient of correlation. These findings suggest that LC neurons of females are more sensitive than those of males to trigeminal imbalance, but also to other afferent signals, thus reducing the correlation between anisocoria and EMG asymmetry. This difference could be attributable to sex-related differences in the afferent pathways to LC neurons or else to LC neuron sensitivity to afferent input [46]. 

### 4.2. Chewing-Induced Stimulation of Visuospatial Performance

The present study investigated the changes in visuospatial performance and pupil size elicited in the same subjects by three different chewing patterns. The results confirmed that unilateral chewing is more effective in enhancing performance when it occurs on the hypotonic side. PI values, however, were significantly increased by chewing on the hypotonic side, at variance with previous investigations [34]. This discrepancy was due to the inclusion of BAL subjects in the present study, while they were absent in previous ones. This indicates that, at variance with IMB subjects who are sensitive only to hypotonic side chewing, BAL individuals derive benefit from chewing independent of the mastication side.

The improvements in cognitive visuospatial performance observed in the present study could be attributed to the increased arousal level induced by the strong connections between the trigeminal system and the ascending reticular activating system (ARAS) [18,47,48], with particular reference to the LC. Sensorimotor trigeminal signals elicited by chewing are likely to modify the activity of LC and its excitability, so that larger mydriasis (i.e., higher LC activation) occurs during the matrices test following the chewing sequence, thus enhancing cognitive visuospatial performance. Although in the present study the mydriasis elicited during the matrices test was not measured, indirect evidence for LC excitability changes may arise from the changes in pupil size. This was the case for hypotonic side chewing, where PI changes could be predicted by pupil size changes observed on the hypotonic side, independent of sex and asymmetry group. Under the hypertonic side chewing condition, which affected PI only in BAL subjects, PI changes were correlated with pupil size changes on the hypertonic side only within this restricted population. The relationship between PI and pupil size changes was less close under the bilateral chewing condition, when the two pupils underwent opposite changes in size. Moreover, this correlation mainly characterized the female group, thus suggesting that pupil size changes in this condition are not well suited to predicting the changes in LC excitability that likely occur following chewing (see Section 4.4). 

The results of the present study indicated that the effects on performance in a cognitive visual search task elicited by two sequential bouts of chewing on the left and right sides cannot be predicted by assuming a simple additive effect of the two chewing periods (predicted value). The difference between the predicted and experimental values was significant whatever the gender and level of trigeminal balance/imbalance. The observed difference could be due to the presence of a build-up of excitation that progressively increased the stimulating effect of chewing on cognitive visuospatial performance. Further studies are required to verify this hypothesis. A well-documented example of brainstem build-up phenomenon is the velocity storage mechanism [49], which progressively increases the velocity of slow compensatory eye movements during optokinetic nystagmus and involves vestibular, cerebellar, and other brainstem structures [50,51]. There is indeed evidence that, at least in pathological conditions, chewing may activate this circuit, giving rise to eye movement of progressively higher velocity [52]. To the best of our knowledge, there is, at present, no evidence that the velocity storage mechanism is implicated in the control of cognitive performance. It is possible, however, that under physiological conditions, trigeminal signals activate other networks during chewing, progressively enhancing ARAS excitability. Whichever mechanism might be involved, its effectiveness seems to be biased by the presence of an LC imbalance, since the difference between the experimental and predicted PI changes observed following bilateral chewing in males was larger in BAL versus IMB subjects. The decay of this build-up phenomenon, more effective in BAL than in IMB subjects, could be also responsible for the larger drop in PI from T1 to T2 observed in BAL with respect to IMB subjects (Figure 6). 

### 4.3. Chewing-Induced Stimulation of Visuospatial Performance and Trigeminal Asymmetries

The present study showed that, in males, trigeminal asymmetries and the related anisocoria values accounted for a sizable fraction of the variability observed in the chewing-related changes in PI, as well as in the relative efficacy of the different chewing patterns in enhancing visuospatial attention. Also, the decay of the effect of bilateral chewing over time (from T1 to T2) was related to the asymmetry indices (Figure 6). These correlations were not observed among females. It must be pointed out that the female sample analyzed in the present study did not include subjects with EMG asymmetries lower than 20%. This might have obscured the detection of possible correlations of PI changes with asymmetry measures. Further experiments are necessary to clarify this point. 

At least among males, however, the stimulating effect of trigeminal sensorimotor signals elicited by chewing was modulated by the level of trigeminal/LC asymmetry, which made the hypertonic side of IMB subjects somehow refractory to chewing-related activation [34]. In addition, cognitive visuospatial performance could be worsened in IMB subjects by a higher level of stress [53,54,55], likely due the occlusal disharmony [56] associated with trigeminal imbalance.

The refractoriness of the hypertonic side to chewing stimulation could be due to too high LC activity on that side. It is well known that cognitive performance/task-related mydriasis are related to the basal level of LC activity [3,4]; when the LC spiking rate is too high (and pupil size too large), task-related mydriasis, which is due to the activation of LC neurons during the task from frontal cortical structures [3,57], and cognitive performance are likely to be depressed. It may well be that, through a similar mechanism, a high level of LC activity (inferred from the large pupil size) on the hypertonic side blunts the responsiveness of the corresponding neurons to chewing-elicited trigeminal signals and impedes the changes in neuronal excitability that might enhance task-related mydriasis following the chewing activity. According to this hypothesis, the increase in pupil size, observed following ipsilateral chewing, was lower on the hypertonic with respect to the hypotonic side [34]. It has been proposed that this refractoriness, associated with high tonic LC discharge on the hypertonic side, could be considered as a preliminary step in the long process that links LC hyperactivity to neurodegeneration [58]. 

The higher sensitivity of BAL subjects to bilateral chewing can be attributed to the fact that their visuospatial performance was affected by chewing on either side and they were more sensitive to the time-cumulative effects of chewing stimulation. Why the hypotonic side in IMB subjects is more sensitive to unilateral chewing than in BAL subjects needs to be explained. It is possible that, in IMB individuals, the persistence of a low resting level of LC activity on this side leads to an enhancement of its excitability following stimulation of trigeminal afferents. Alternatively, we must consider that chewing on the hypotonic side reduces anisocoria in IMB subjects, which might induce enhanced performance in these subjects. Indeed, as proposed by Tramonti Fantozzi and colleagues [29,30], the presence of anisocoria, i.e., an imbalance in the systems controlling arousal and pupil size (namely the LC), may, in turn, introduce asymmetries in hemispheric excitability. This may lead to cognitive impairment, as suggested by the effects of bilateral and unilateral lesions in animal experiments [59]. Indeed, occlusal corrections attenuating EMG asymmetries and increasing visuospatial performance also greatly decrease anisocoria [28,29,30]. The correlation observed between changes in PI and anisocoria under the hypotonic side chewing condition are consistent with this hypothesis.

Overall, these findings suggest that, in males, the level of trigeminal asymmetry determines the degree of LC imbalance, which finely modulates the effectiveness of chewing-related trigeminal signals in facilitating cognitive visuospatial improvements in a side-dependent manner.

### 4.4. Chewing-Induced Pupil Size Changes

The present investigation showed that, at variance with unilateral chewing, which increased pupil size on both sides [34], bilateral chewing increased pupil size only on the hypotonic side while decreasing it on the hypertonic side, leading to a significant decrease in anisocoria values in IMB subjects.

The difference observed between the hypertonic and hypotonic side pupils during bilateral chewing could arise from the interaction of two opposing factors. Prolonged chewing activity might reduce the level of stress in subjects [60,61], reducing EMG activity, LC discharge, and pupil size. In addition, sensorimotor trigeminal activity during chewing might increase LC activity (and pupil size), leading to a larger pupil diameter following the chewing period [34]. In the present and earlier studies [34], unilateral chewing lasted for one minute only, while bilateral chewing lasted for two minutes. This may have favored the effect of reduced stress level, in the bilateral chewing condition, leading to a reduction of baseline LC activity on the hypertonic side. According to this hypothesis, the increase in pupil size observed on the hypotonic side following bilateral chewing was significantly less than that observed following chewing on the hypotonic side (Table S1).

The pupil size changes, elicited by chewing on the hypotonic side in the ipsilateral pupil, were tuned by EMG asymmetry (and anisocoria) so that the larger post-chewing increases in pupil size occurred in the subjects showing the largest asymmetry values. Also, this finding was consistent with a higher sensitivity of the LC neurons on the hypotonic side to trigeminal stimulation in IMB subjects, leading to a slightly higher LC discharge and pupil size soon after the chewing activity. Assuming that this larger pupil size is indicative of higher excitability of LC neurons during the matrices test and, consequently, of larger task-related mydriasis, this would explain the higher sensitivity of PI to hypotonic side chewing in IMB subjects. This higher sensitivity of the LC on the hypotonic side in IMB subjects would also explain the larger drop in anisocoria values of these subjects, which may further enhance chewing-elicited visuospatial improvement. 

Finally, the present experiments showed that a drop in anisocoria values occurred also following bilateral chewing, which was larger in subjects showing the highest asymmetry values. This might have improved the sensitivity of IMB subjects to bilateral chewing. 

### 4.5. Neurophysiological Mechanisms

The neurophysiological mechanisms responsible for these changes in neuronal excitability following chewing (and the lack thereof when chewing occurs unilaterally on the hypertonic side) are unknown. Following bilateral chewing, no increase in pupil size at rest was observed, suggesting that no relevant changes occurred in the tonic LC activity of both sides. However, larger task-related mydriasis was induced following chewing activity [19], indicating that the LC is more responsive to inputs from supratentorial structures [3,57] during cognitive tasks. These changes in LC excitability may be due to specific neurotransmitters/modulators acting on specific channels and receptors expressed on the LC neurons, such as the G protein-coupled inwardly rectifying and small conductance calcium-activated potassium channels, as well as the α2 adrenergic receptor [62,63,64,65,66]. Moreover, an excitatory neurotransmitter such as orexin could be released in the LC following trigeminal stimulation [67,68,69,70], thus increasing LC neuron discharge synchrony [71], while afferent stimulations increase the expression of connexins within the LC [72]. Any mechanisms involved, however, must induce persistent effects on LC excitability that last up to 30 minutes following (bilateral) chewing activity.

### 4.6. Conclusions

In conclusion, the present study showed that BAL subjects benefitted from the stimulating effects of unilateral chewing irrespective of chewing side and were more sensitive than IMB subjects to bilateral chewing. Moreover, the effects of bilateral chewing could not be predicted by summing those elicited by unilateral chewing on both sides, suggesting that prolonged chewing activity leads to a build-up of excitation within the central structures that mediates the stimulating effect of chewing on performance of a visual attention task. BAL subjects were more sensitive than IMB subjects to the stimulating effects of bilateral chewing, likely due to the effectiveness of both sides in activating ARAS during chewing and a higher ability to recruit a build-up of excitation within brainstem structures. Finally, in males, the changes in visuospatial performance elicited under all chewing conditions, as well as their relative efficacy, were tuned by the degree of trigeminal asymmetry and anisocoria. 

### 4.7. Translational Applications

The present investigation explores the influence of chewing on visuospatial attention and its interaction with the presence of basal trigeminal asymmetry, which are coupled to an apparent imbalance of the central noradrenergic system. There are two possible translational applications arising from this study and previous ones targeting the same topic [28,29,30,32,34]. First of all, coupling a chewing training protocol to correction of the trigeminal imbalance could facilitate the cognitive and possibly motor performance [32] of individual subjects. 

The second aspect may concern the field of rehabilitation. There is growing evidence that neurodegenerative diseases may be triggered by neurodegenerative processes occurring in the LC [73,74]. In parallel, it is well known that a masticatory deficit may lead to brain neurodegenerative processes [18,74,75]. Given the coupling between the trigeminal system and LC, there is the intriguing possibility that a disruption of trigeminal input due to occlusal pathologies may play a role in neurodegenerative disease. In this respect, oral rehabilitation could be proposed as a therapeutic strategy for improving some aspect of a patient’s condition. Anecdotal observations in patients affected by neurodegenerative diseases [76] undergoing the same occlusal rebalancing procedure described in the present study indicate the occurrence of cognitive and motor improvements.

Further studies are needed to verify the suitability of chewing and occlusal rebalancing within these two promising fields of application. 

### 4.8. Limitations of the Study

We must acknowledge that this study had several limitations. First, the sample size was rather small. This limited the possibly of detecting significant correlations within the individual male and female populations. Moreover, all of the females were IMB subjects, thus making it impossible to draw final conclusions about the lack of correlations between pupil size/performance changes and asymmetry indices within this gender group.

## Figures and Tables

**Table 1:** Mean ± SD values of performance index (PI), hypertonic and hypotonic pupil size, and anisocoria observed at various time points (T0, T1, T2). The results of a 3 Times (T0, T1, T2) repeated measures ANOVA and relative post hoc values are reported. A. Bilateral chewing. B. Chewing on the hypertonic side. C. Chewing on the hypotonic side. D. Rest. The significance level corresponds to 0.05 for PI. Since pupil size and anisocoria were considered as non-independent variables, comparisons between time points and F values were submitted to Bonferroni’s correction and the significance level corresponds to 0.017. NS: Not Significant.

		Time Points and Post Hoc Comparisons						
Condition	Parameter	T0	T1 vs. T0	T1	T1 vs. T2	T2	T2 vs. T0	ANOVA
**A. Bilateral Chewing**	**Performance Index** **(PI, Nos./s)**	1.78 ± 0.49	*p* < 0.0005	2.40 ± 0.59	*p* = 0.001	2.25 ± 0.54	*p* < 0.0005	F(2,58) = 128.49 *p* < 0.0005 η^2^ = 0.816
	**Pupil Size, Hypertonic side (mm)**	3.99 ± 0.81	*p* = 0.009	3.86 ± 0.77	NS	3.88 ± 0.67	NS	F(2,58) = 5.512 *p* = 0.006 η^2^ = 0.160
	**Pupil Size, Hypotonic side (mm)**	3.67 ± 0.81	*p* = 0.002	3.81 ± 0.71	NS	3.77 ± 0.71	NS	F(2,58) = 6.81 *p* = 0.001 η^2^ = 0.190
	**Anisocoria (mm)**	0.32 ± 0.32	*p* < 0.0005	0.06 ± 0.22	NS	0.12 ± 0.19	*p* = 0.001	F(2,58) = 17.22 *p* < 0.0005 η^2^ = 0.373
**B. Chewing on the Hypertonic side**	**Performance Index** **(PI, Nos./s)**	1.71 ± 0.48	*p* < 0.0005	1.78 ± 0.48	*p* = 0.037	1.73 ± 0.48	*p* = 0.004	F(2,58) = 14.24 *p* < 0.0005 η^2^ = 0.331
	**Pupil Size, Hypertonic side (mm)**	4.04 ± 0.79	*p* < 0.0005	4.23 ± 0.74	*p* < 0.0005	4.12 ± 0.80	*p* < 0.0005	F(2,58) = 63.60 *p* < 0.0005 η^2^ = 0.687
	**Pupil Size, Hypotonic side (mm)**	3.68 ± 0.71	*p* < 0.0005	3.74 ± 0.72	*p* = 0.006	3.70 ± 0.73	*p* = 0.013	F(2,58) = 17.84 *p* < 0.0005 η^2^ = 0.381
	**Anisocoria (mm)**	0.37 ± 0.31	*p* < 0.0005	0.49 ± 0.29	*p* = 0.010	0.42 ± 0.34	*p* = 0.004	F(2,58) = 18.08 *p* < 0.0005 η^2^ = 0.384
**C. Chewing on the Hypotonic side**	**Performance Index** **(PI, Nos./s)**	1.73 ± 0.47	*p* < 0.0005	1.95 ± 0.49	*p* < 0.0005	1.77 ± 0.44	*p* = 0.013	F(2,58) = 63.67 *p* < 0.0005 η^2^ = 0.687
	**Pupil Size, Hypertonic side (mm)**	4.04 ± 0.79	*p* < 0.0005	4.09 ± 0.79	NS	4.08 ± 0.79	*p* = 0.003	F(2,58) = 13.25 *p* < 0.0005 η^2^ = 0.314
	**Pupil Size, Hypotonic side (mm)**	3.71 ± 0.70	*p* < 0.0005	3.99 ± 0.73	*p* < 0.0005	3.76 ± 0.71	NS	F(2,58) = 91.31 *p* < 0.0005 η^2^ = 0.759
	**Anisocoria (mm)**	0.33 ± 0.33	*p* < 0.0005	0.10 ± 0.28	*p* < 0.0005	0.33 ± 0.34	NS	F(2,58) = 57.53 *p* < 0.0005 η^2^ = 0.665
**D. Rest**	**Performance Index** **(PI, Nos./s)**	1.71 ± 0.52	NS	1.76 ± 0.54	NS	1.76 ± 0.74	NS	F(2,58) = 2.77 NS η^2^ = 0.087
	**Pupil Size, Hypertonic side (mm)**	4.01 ± 0.81	NS	4.05 ± 0.81	NS	4.03 ± 0.80	NS	F(2,58) = 1.08 NS η^2^ = 0.036
	**Pupil Size, Hypotonic side (mm)**	3.74 ± 0.72	NS	3.71 ± 0.73	NS	3.77 ± 0.75	NS	F(2,58) = 2.34 NS η^2^ = 0.072
	**Anisocoria** **(mm)**	0.27 ± 0.27	*p* = 0.016	0.34 ± 0.31	NS	0.26 ± 0.31	NS	F(2,58) = 3.711 NS η^2^ = 0.113

## Data Availability

The data will be made publicly available upon publication in the Open Science Framework (OSF) and a link will be provided.

## Supplementary Materials

The following supporting information can be downloaded at: https://www.mdpi.com/article/10.3390/biomedicines11082307/s1, Table S1: Mean ± SD values of the changes in Performance Index (PI), pupil size and anisocoria observed at T1 and T2 with respect to T0 in four different conditions.
