# Supplementary material for: Trigeminal Stimulation and Visuospatial Performance: The Struggle between Chewing and Trigeminal Asymmetries
Source: Biomedicines. 2023 Aug 19;11(8):2307. doi: 10.3390/biomedicines11082307 (PMC10452603; doi:10.3390/biomedicines11082307)
Supplement: Supplementary file 1 [file biomedicines-11-02307-s001.zip › biomedicines-2522652-supplemetary material-update.pdf]

Parameter	Time Point	A. Bilateral Chewing	B. Chewing on Hypertonic side	C. Chewing on Hypotonic side	D. Rest	A vs B	A vs C	B vs C	A vs D	B vs D	C vs D
Performance Index (PI, Nos./s)	T1	0.62±0.25*	0.07±0.08*	0.21±0.12*	0.04±0.14	p<0.0005	p<0.0005	p<0.0005	p<0.0005	NS	p<0.0005
	T1 vs T2	p=0.001	p=0.004	p<0.0005	NS						
	T2	0.47±0.18*	0.02±0.05*	0.04±0.08*	0.04±0.012	p<0.0005	p<0.0005	NS	p<0.0005	NS	NS
Pupil Size, Hypertonic side (mm)	T1	-0.12±0.24*	0.18±0.08*	0.05±0.03*	0.04±0.16	p<0.0005	p<0.0005	p<0.0005	p=0.010	p<0.0005	NS
	T1 vs T2	NS	p<0.0005	NS	NS						
	T2	-0.10±0.24	0.08±0.07*	0.05±0.08*	0.02±0.12	p<0.0005	p=0.002	NS	p=0.015	NS	NS
Pupil Size, Hypotonic side (mm)	T1	0.14±0.22*	0.06±0.05*	0.28±0.09*	-0.03±0.16	NS	p=0.001	p<0.0005	p=0.007	p=0.009	p<0.0005
	T1 vs T2	NS	p=0.006	p<0.0005	NS						
	T2	0.10±0.24	0.02±0.05*	0.05±0.12	0.03±0.16	NS	NS	NS	NS	NS	NS
Anisocoria (mm)	T1	-0.26±0.28*	0.12±0.10*	-0.23±0.10*	0.07±0.15*	p<0.0005	NS	p<0.0005	p<0.0005	NS	p<0.0005
	T1 vs T2	NS	p=0.010	p<0.005	NS						
	T2	-0.20±0.29*	0.05±0.09*	0.00±0.14	0.00±0.17	p<0.0001	p=0.002	NS	p=0.004	NS	NS

Table S1 Mean±SD values of the changes in Performance Index (PI), pupil size and anisocoria observed at T1 and T2 with respect to T0 in four different conditions. (A: Bilateral Chewing; B: Chewing on Hypertonic side; C: Chewing on Hypotonic side; D: Rest). Comparison between conditions and time points have been reported in columns and lines, respectively. The significance level corresponded to 0.05 for PI. Since pupil size and anisocoria were considered as non-independent variables, comparison between time points and F values were submitted to Bonferroni's correction and the significance level corresponded to 0.017. The asterisks indicate statistically significant difference between the corresponding time point and T0. NS: Not Significant.
